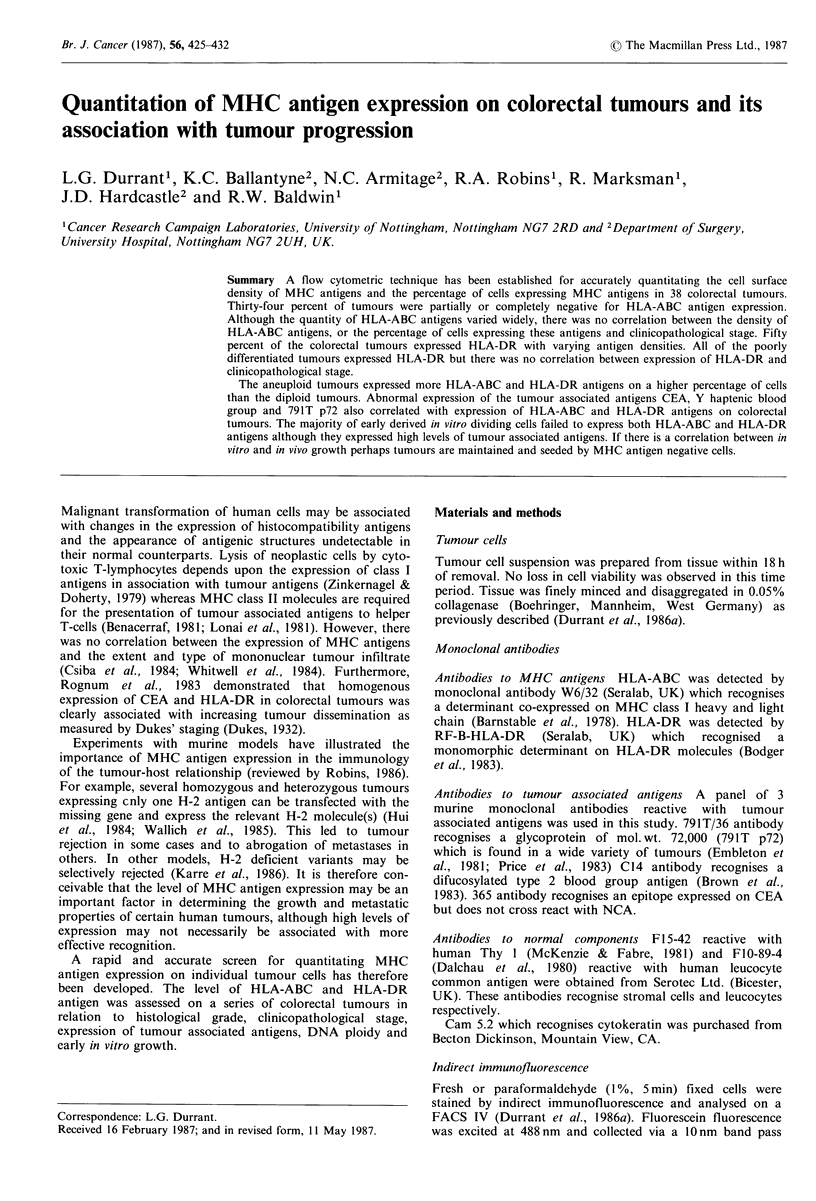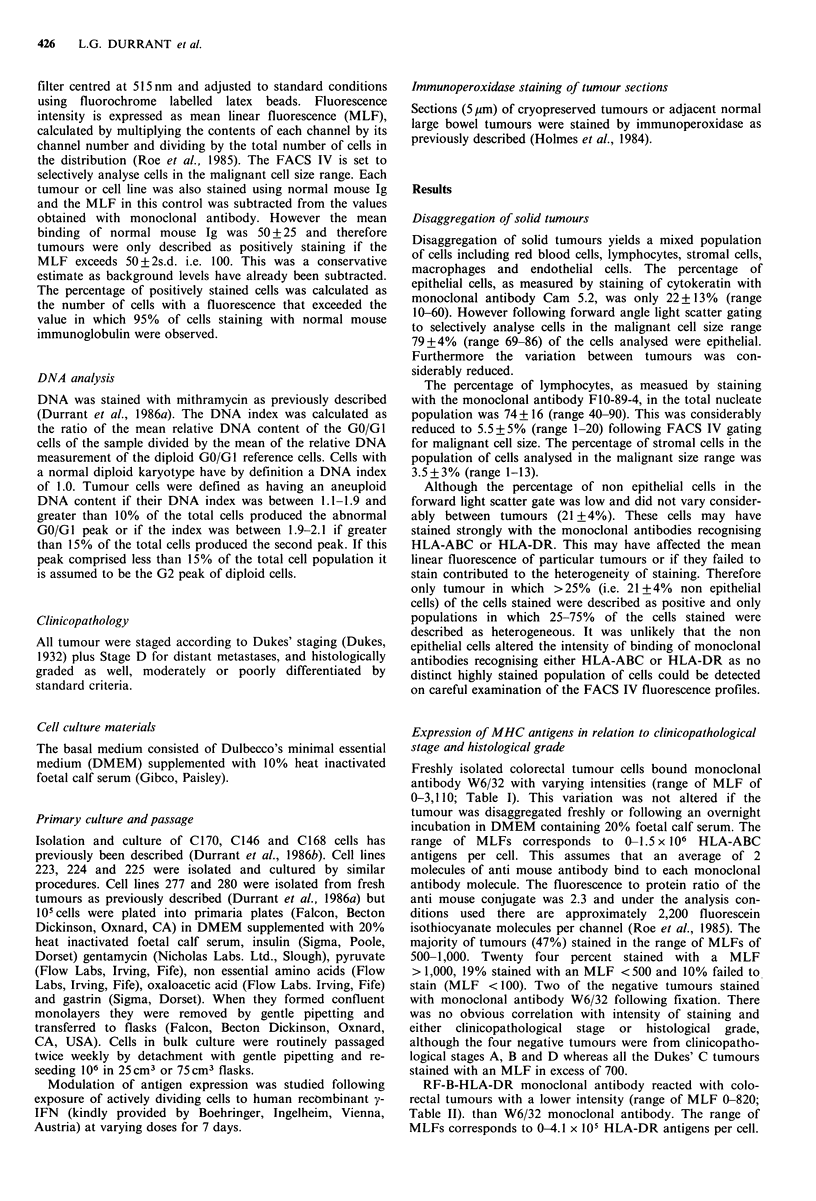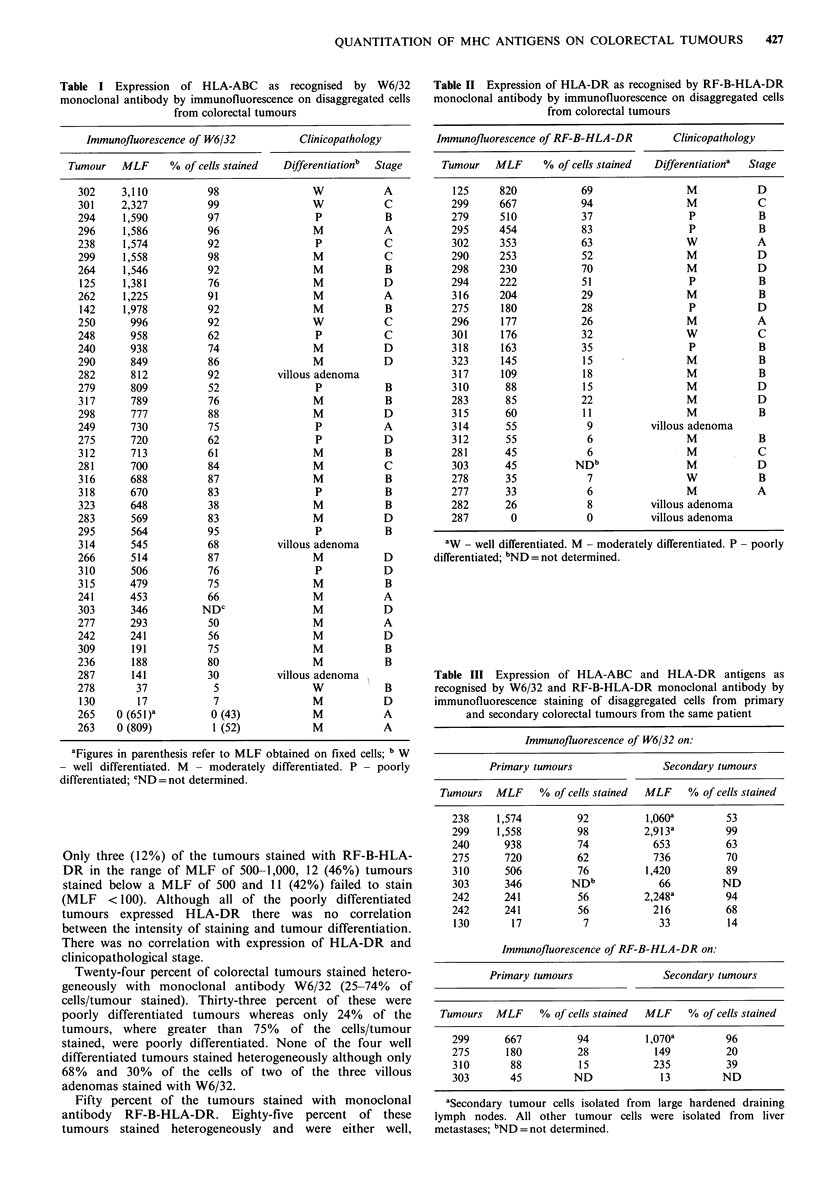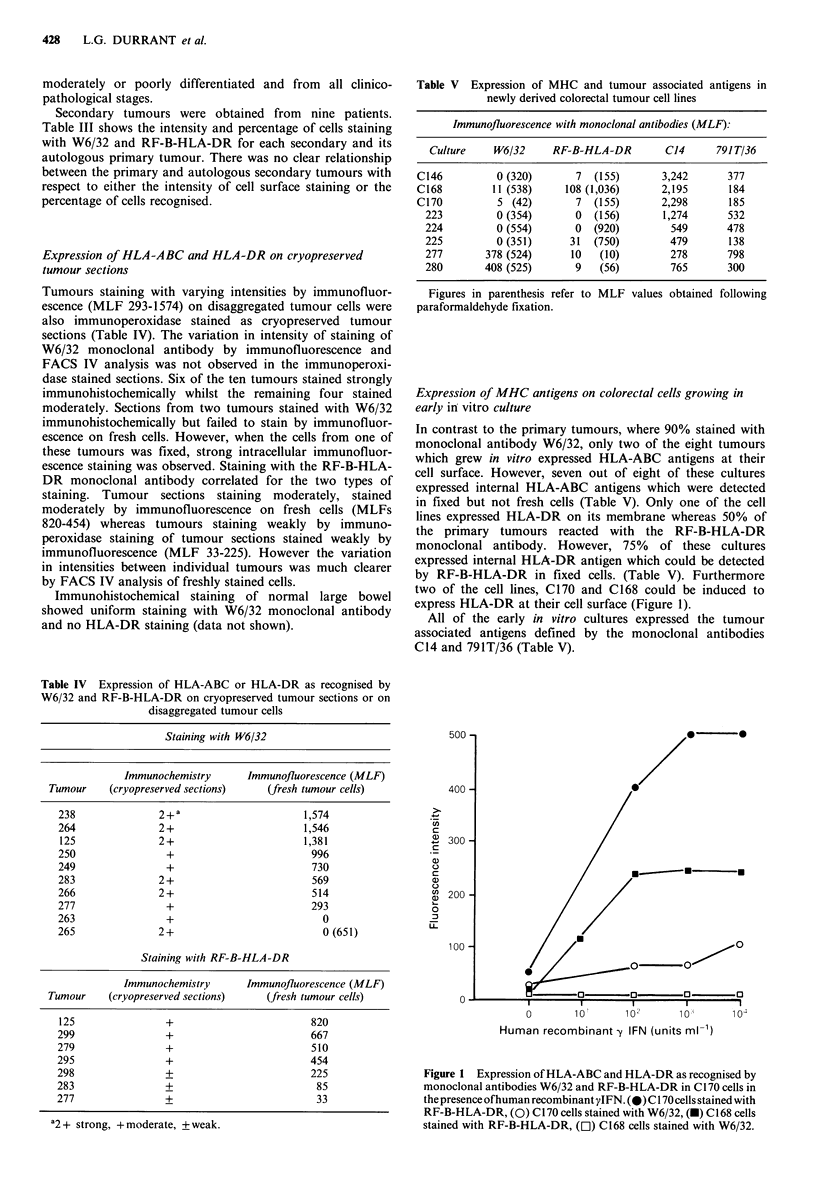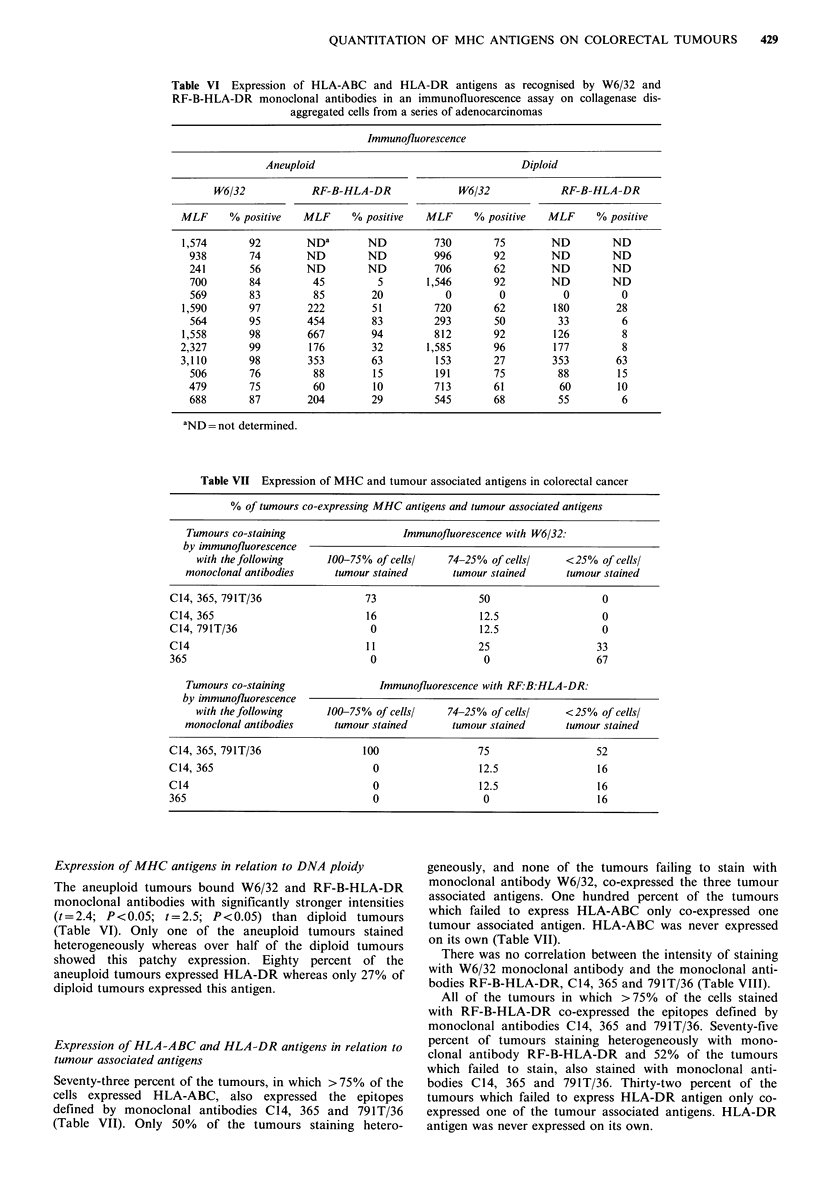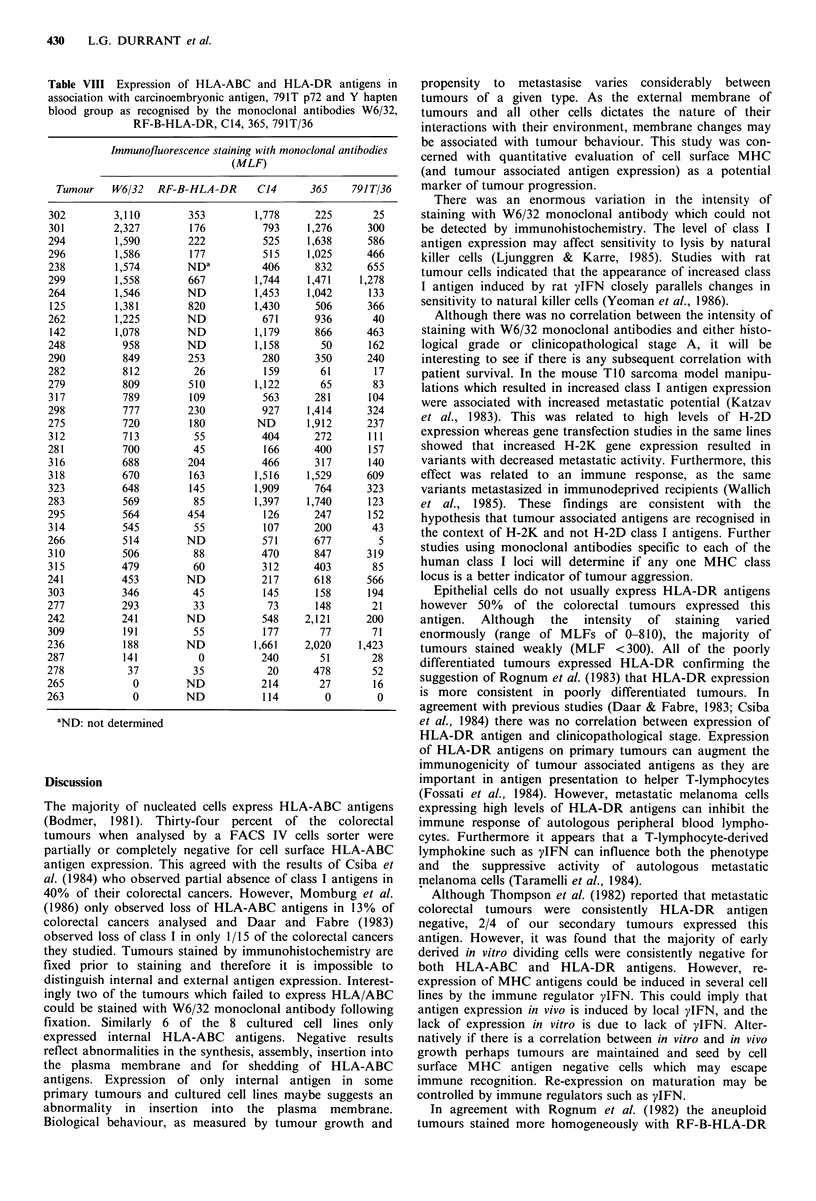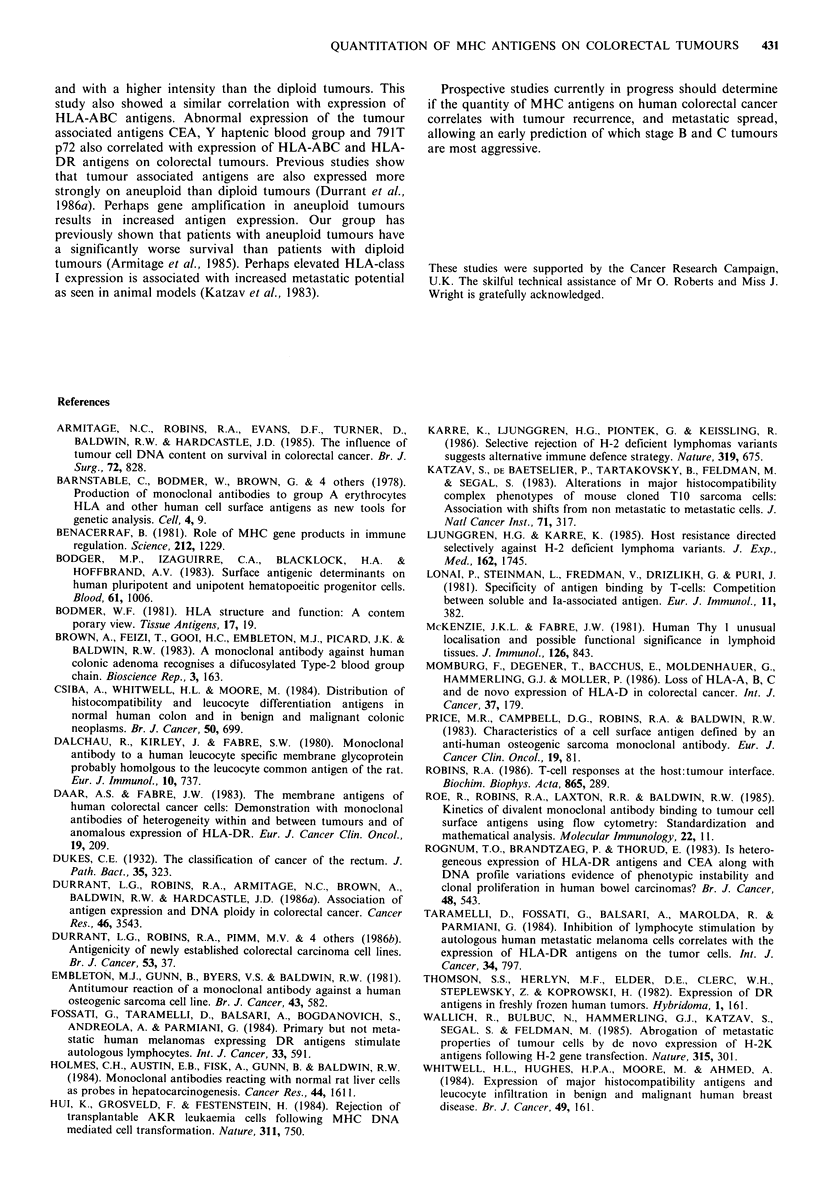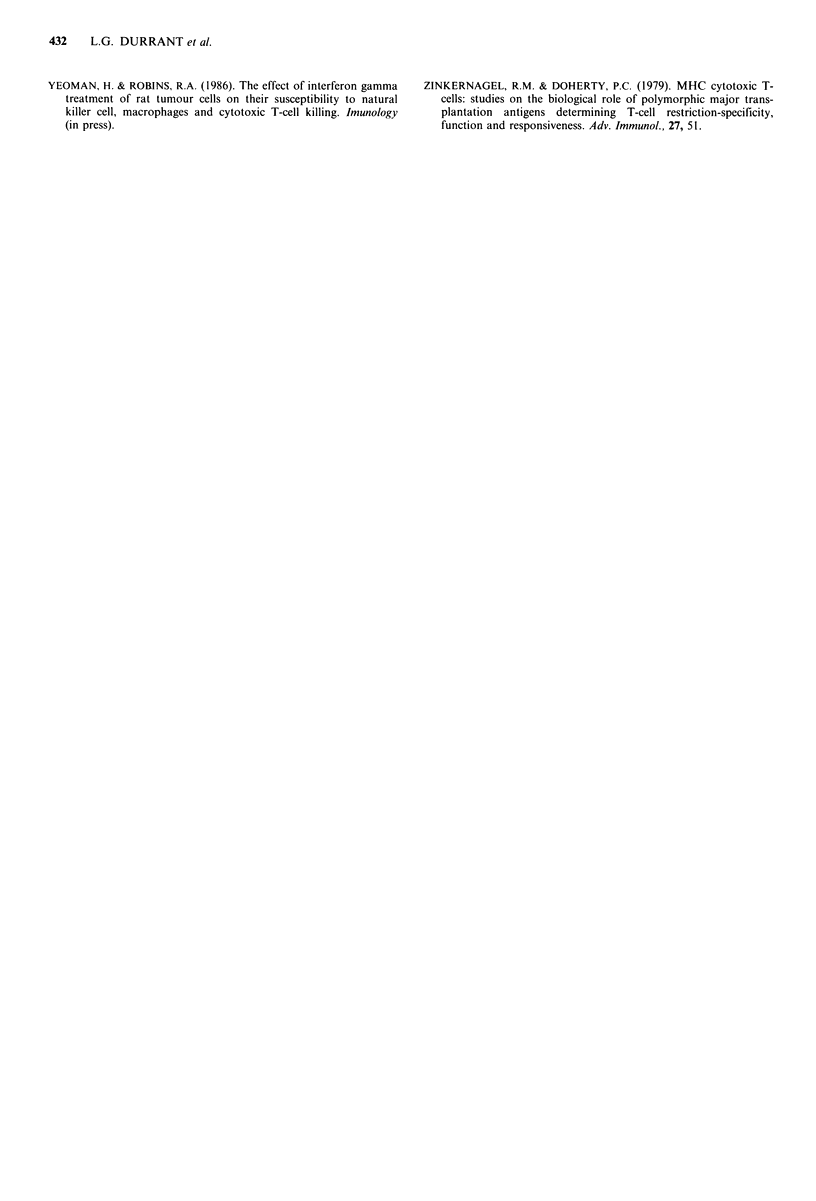# Quantitation of MHC antigen expression on colorectal tumours and its association with tumour progression.

**DOI:** 10.1038/bjc.1987.218

**Published:** 1987-10

**Authors:** L. G. Durrant, K. C. Ballantyne, N. C. Armitage, R. A. Robins, R. Marksman, J. D. Hardcastle, R. W. Baldwin

**Affiliations:** Cancer Research Campaign Laboratories, University of Nottingham, UK.

## Abstract

A flow cytometric technique has been established for accurately quantitating the cell surface density of MHC antigens and the percentage of cells expressing MHC antigens in 38 colorectal tumours. Thirty-four percent of tumours were partially or completely negative for HLA-ABC antigen expression. Although the quantity of HLA-ABC antigens varied widely, there was no correlation between the density of HLA-ABC antigens, or the percentage of cells expressing these antigens and clinicopathological stage. Fifty percent of the colorectal tumours expressed HLA-DR with varying antigen densities. All of the poorly differentiated tumours expressed HLA-DR but there was no correlation between expression of HLA-DR and clinicopathological stage. The aneuploid tumours expressed more HLA-ABC and HLA-DR antigens on a higher percentage of cells than the diploid tumours. Abnormal expression of the tumour associated antigens CEA, Y haptenic blood group and 791T p72 also correlated with expression of HLA-ABC and HLA-DR antigens on colorectal tumours. The majority of early derived in vitro dividing cells failed to express both HLA-ABC and HLA-DR antigens although they expressed high levels of tumour associated antigens. If there is a correlation between in vitro and in vivo growth perhaps tumours are maintained and seeded by MHC antigen negative cells.


*Br. J. Cancer* (1987), **56**, 425–432 © The Macmillan Press Ltd., 1987

### Quantitation of MHC antigen expression on colorectal tumours and its association with tumour progression

L.G. Durrant[1], K.C. Ballantyne[2], N.C. Armitage[2], R.A. Robins[1], R. Marksman[1], J.D. Hardcastle[2] and R.W. Baldwin[1]

[1]*Cancer Research Campaign Laboratories, University of Nottingham, Nottingham NG7 2RD and* [2]*Department of Surgery, University Hospital, Nottingham NG7 2UH, UK.*

**Summary** A flow cytometric technique has been established for accurately quantitating the cell surface density of MHC antigens and the percentage of cells expressing MHC antigens in 38 colorectal tumours. Thirty-four percent of tumours were partially or completely negative for HLA-ABC antigen expression. Although the quantity of HLA-ABC antigens varied widely, there was no correlation between the density of HLA-ABC antigens, or the percentage of cells expressing these antigens and clinicopathological stage. Fifty percent of the colorectal tumours expressed HLA-DR with varying antigen densities. All of the poorly differentiated tumours expressed HLA-DR but there was no correlation between expression of HLA-DR and clinicopathological stage.

The aneuploid tumours expressed more HLA-ABC and HLA-DR antigens on a higher percentage of cells than the diploid tumours. Abnormal expression of the tumour associated antigens CEA, Y haptenic blood group and 791T p72 also correlated with expression of HLA-ABC and HLA-DR antigens on colorectal tumours. The majority of early derived *in vitro* dividing cells failed to express both HLA-ABC and HLA-DR antigens although they expressed high levels of tumour associated antigens. If there is a correlation between *in vitro* and *in vivo* growth perhaps tumours are maintained and seeded by MHC antigen negative cells.

Malignant transformation of human cells may be associated with changes in the expression of histocompatibility antigens and the appearance of antigenic structures undetectable in their normal counterparts. Lysis of neoplastic cells by cytotoxic T-lymphocytes depends upon the expression of class I antigens in association with tumour antigens (Zinkernagel & Doherty, 1979) whereas MHC class II molecules are required for the presentation of tumour associated antigens to helper T-cells (Benacerraf, 1981; Lonai *et al.*, 1981). However, there was no correlation between the expression of MHC antigens and the extent and type of mononuclear tumour infiltrate (Csiba *et al.*, 1984; Whitwell *et al.*, 1984). Furthermore, Rognum *et al.*, 1983 demonstrated that homogenous expression of CEA and HLA-DR in colorectal tumours was clearly associated with increasing tumour dissemination as measured by Dukes' staging (Dukes, 1932).

Experiments with murine models have illustrated the importance of MHC antigen expression in the immunology of the tumour-host relationship (reviewed by Robins, 1986). For example, several homozygous and heterozygous tumours expressing cnly one H-2 antigen can be transfected with the missing gene and express the relevant H-2 molecule(s) (Hui *et al.*, 1984; Wallich *et al.*, 1985). This led to tumour rejection in some cases and to abrogation of metastases in others. In other models, H-2 deficient variants may be selectively rejected (Karre *et al.*, 1986). It is therefore conceivable that the level of MHC antigen expression may be an important factor in determining the growth and metastatic properties of certain human tumours, although high levels of expression may not necessarily be associated with more effective recognition.

A rapid and accurate screen for quantitating MHC antigen expression on individual tumour cells has therefore been developed. The level of HLA-ABC and HLA-DR antigen was assessed on a series of colorectal tumours in relation to histological grade, clinicopathological stage, expression of tumour associated antigens, DNA ploidy and early *in vitro* growth.

Correspondence: L.G. Durrant.
Received 16 February 1987; and in revised form, 11 May 1987.

### Materials and methods

### *Tumour cells*

Tumour cell suspension was prepared from tissue within 18 h of removal. No loss in cell viability was observed in this time period. Tissue was finely minced and disaggregated in 0.05% collagenase (Boehringer, Mannheim, West Germany) as previously described (Durrant *et al.*, 1986*a*).

### *Monoclonal antibodies*

*Antibodies to MHC antigens* HLA-ABC was detected by monoclonal antibody W6/32 (Seralab, UK) which recognises a determinant co-expressed on MHC class I heavy and light chain (Barnstable *et al.*, 1978). HLA-DR was detected by RF-B-HLA-DR (Seralab, UK) which recognised a monomorphic determinant on HLA-DR molecules (Bodger *et al.*, 1983).

*Antibodies to tumour associated antigens* A panel of 3 murine monoclonal antibodies reactive with tumour associated antigens was used in this study. 791T/36 antibody recognises a glycoprotein of mol. wt. 72,000 (791T p72) which is found in a wide variety of tumours (Embleton *et al.*, 1981; Price *et al.*, 1983) C14 antibody recognises a difucosylated type 2 blood group antigen (Brown *et al.*, 1983). 365 antibody recognises an epitope expressed on CEA but does not cross react with NCA.

*Antibodies to normal components* F15-42 reactive with human Thy 1 (McKenzie & Fabre, 1981) and F10-89-4 (Dalchau *et al.*, 1980) reactive with human leucocyte common antigen were obtained from Serotec Ltd. (Bicester, UK). These antibodies recognise stromal cells and leucocytes respectively.

Cam 5.2 which recognises cytokeratin was purchased from Becton Dickinson, Mountain View, CA.

### *Indirect immunofluorescence*

Fresh or paraformaldehyde (1%, 5 min) fixed cells were stained by indirect immunofluorescence and analysed on a FACS IV (Durrant *et al.*, 1986*a*). Fluorescein fluorescence was excited at 488 nm and collected via a 10 nm band pass filter centred at 515 nm and adjusted to standard conditions using fluorochrome labelled latex beads. Fluorescence intensity is expressed as mean linear fluorescence (MLF), calculated by multiplying the contents of each channel by its channel number and dividing by the total number of cells in the distribution (Roe *et al.*, 1985). The FACS IV is set to selectively analyse cells in the malignant cell size range. Each tumour or cell line was also stained using normal mouse Ig and the MLF in this control was subtracted from the values obtained with monoclonal antibody. However the mean binding of normal mouse Ig was $50 \pm 25$ and therefore tumours were only described as positively staining if the MLF exceeds $50 \pm 2$s.d. i.e. 100. This was a conservative estimate as background levels have already been subtracted. The percentage of positively stained cells was calculated as the number of cells with a fluorescence that exceeded the value in which 95% of cells staining with normal mouse immunoglobulin were observed.

### *DNA analysis*

DNA was stained with mithramycin as previously described (Durrant *et al.*, 1986*a*). The DNA index was calculated as the ratio of the mean relative DNA content of the G0/G1 cells of the sample divided by the mean of the relative DNA measurement of the diploid G0/G1 reference cells. Cells with a normal diploid karyotype have by definition a DNA index of 1.0. Tumour cells were defined as having an aneuploid DNA content if their DNA index was between 1.1–1.9 and greater than 10% of the total cells produced the abnormal G0/G1 peak or if the index was between 1.9–2.1 if greater than 15% of the total cells produced the second peak. If this peak comprised less than 15% of the total cell population it is assumed to be the G2 peak of diploid cells.

### *Clinicopathology*

All tumour were staged according to Dukes' staging (Dukes, 1932) plus Stage D for distant metastases, and histologically graded as well, moderately or poorly differentiated by standard criteria.

### *Cell culture materials*

The basal medium consisted of Dulbecco's minimal essential medium (DMEM) supplemented with 10% heat inactivated foetal calf serum (Gibco, Paisley).

### *Primary culture and passage*

Isolation and culture of C170, C146 and C168 cells has previously been described (Durrant *et al.*, 1986*b*). Cell lines 223, 224 and 225 were isolated and cultured by similar procedures. Cell lines 277 and 280 were isolated from fresh tumours as previously described (Durrant *et al.*, 1986*a*) but $10^5$ cells were plated into primaria plates (Falcon, Becton Dickinson, Oxnard, CA) in DMEM supplemented with 20% heat inactivated foetal calf serum, insulin (Sigma, Poole, Dorset) gentamycin (Nicholas Labs. Ltd., Slough), pyruvate (Flow Labs, Irving, Fife), non essential amino acids (Flow Labs, Irving, Fife), oxaloacetic acid (Flow Labs. Irving, Fife) and gastrin (Sigma, Dorset). When they formed confluent monolayers they were removed by gentle pipetting and transferred to flasks (Falcon, Becton Dickinson, Oxnard, CA, USA). Cells in bulk culture were routinely passaged twice weekly by detachment with gentle pipetting and re-seeding $10^6$ in 25 cm$^3$ or 75 cm$^3$ flasks.

Modulation of antigen expression was studied following exposure of actively dividing cells to human recombinant $\gamma$-IFN (kindly provided by Boehringer, Ingelheim, Vienna, Austria) at varying doses for 7 days.

### *Immunoperoxidase staining of tumour sections*

Sections (5 $\mu$m) of cryopreserved tumours or adjacent normal large bowel tumours were stained by immunoperoxidase as previously described (Holmes *et al.*, 1984).

### Results

### *Disaggregation of solid tumours*

Disaggregation of solid tumours yields a mixed population of cells including red blood cells, lymphocytes, stromal cells, macrophages and endothelial cells. The percentage of epithelial cells, as measured by staining of cytokeratin with monoclonal antibody Cam 5.2, was only $22 \pm 13$% (range 10–60). However following forward angle light scatter gating to selectively analyse cells in the malignant cell size range $79 \pm 4$% (range 69–86) of the cells analysed were epithelial. Furthermore the variation between tumours was considerably reduced.

The percentage of lymphocytes, as measued by staining with the monoclonal antibody F10-89-4, in the total nucleate population was $74 \pm 16$ (range 40–90). This was considerably reduced to $5.5 \pm 5$% (range 1–20) following FACS IV gating for malignant cell size. The percentage of stromal cells in the population of cells analysed in the malignant size range was $3.5 \pm 3$% (range 1–13).

Although the percentage of non epithelial cells in the forward light scatter gate was low and did not vary considerably between tumours ($21 \pm 4$%). These cells may have stained strongly with the monoclonal antibodies recognising HLA-ABC or HLA-DR. This may have affected the mean linear fluorescence of particular tumours or if they failed to stain contributed to the heterogeneity of staining. Therefore only tumour in which >25% (i.e. $21 \pm 4$% non epithelial cells) of the cells stained were described as positive and only populations in which 25–75% of the cells stained were described as heterogeneous. It was unlikely that the non epithelial cells altered the intensity of binding of monoclonal antibodies recognising either HLA-ABC or HLA-DR as no distinct highly stained population of cells could be detected on careful examination of the FACS IV fluorescence profiles.

### *Expression of MHC antigens in relation to clinicopathological stage and histological grade*

Freshly isolated colorectal tumour cells bound monoclonal antibody W6/32 with varying intensities (range of MLF of 0–3,110; Table I). This variation was not altered if the tumour was disaggregated freshly or following an overnight incubation in DMEM containing 20% foetal calf serum. The range of MLFs corresponds to $0–1.5 \times 10^6$ HLA-ABC antigens per cell. This assumes that an average of 2 molecules of anti mouse antibody bind to each monoclonal antibody molecule. The fluorescence to protein ratio of the anti mouse conjugate was 2.3 and under the analysis conditions used there are approximately 2,200 fluorescein isothiocyanate molecules per channel (Roe *et al.*, 1985). The majority of tumours (47%) stained in the range of MLFs of 500–1,000. Twenty four percent stained with a MLF >1,000, 19% stained with an MLF <500 and 10% failed to stain (MLF <100). Two of the negative tumours stained with monoclonal antibody W6/32 following fixation. There was no obvious correlation with intensity of staining and either clinicopathological stage or histological grade, although the four negative tumours were from clinicopathological stages A, B and D whereas all the Dukes' C tumours stained with an MLF in excess of 700.

RF-B-HLA-DR monoclonal antibody reacted with colorectal tumours with a lower intensity (range of MLF 0–820; Table II). than W6/32 monoclonal antibody. The range of MLFs corresponds to $0–4.1 \times 10^5$ HLA-DR antigens per cell.

**Table I** Expression of HLA-ABC as recognised by W6/32 monoclonal antibody by immunofluorescence on disaggregated cells from colorectal tumours

| *Immunofluorescence of W6/32* | | | *Clinicopathology* | |
|---|---|---|---|---|
| *Tumour* | *MLF* | *% of cells stained* | *Differentiation*[b] | *Stage* |
| 302 | 3,110 | 98 | W | A |
| 301 | 2,327 | 99 | W | C |
| 294 | 1,590 | 97 | P | B |
| 296 | 1,586 | 96 | M | A |
| 238 | 1,574 | 92 | P | C |
| 299 | 1,558 | 98 | M | C |
| 264 | 1,546 | 92 | M | B |
| 125 | 1,381 | 76 | M | D |
| 262 | 1,225 | 91 | M | A |
| 142 | 1,978 | 92 | M | B |
| 250 | 996 | 92 | W | C |
| 248 | 958 | 62 | P | C |
| 240 | 938 | 74 | M | D |
| 290 | 849 | 86 | M | D |
| 282 | 812 | 92 | villous adenoma | |
| 279 | 809 | 52 | P | B |
| 317 | 789 | 76 | M | B |
| 298 | 777 | 88 | M | D |
| 249 | 730 | 75 | P | A |
| 275 | 720 | 62 | P | D |
| 312 | 713 | 61 | M | B |
| 281 | 700 | 84 | M | C |
| 316 | 688 | 87 | M | B |
| 318 | 670 | 83 | P | B |
| 323 | 648 | 38 | M | B |
| 283 | 569 | 83 | M | D |
| 295 | 564 | 95 | P | B |
| 314 | 545 | 68 | villous adenoma | |
| 266 | 514 | 87 | M | D |
| 310 | 506 | 76 | P | D |
| 315 | 479 | 75 | M | B |
| 241 | 453 | 66 | M | A |
| 303 | 346 | ND[c] | M | D |
| 277 | 293 | 50 | M | A |
| 242 | 241 | 56 | M | D |
| 309 | 191 | 75 | M | B |
| 236 | 188 | 80 | M | B |
| 287 | 141 | 30 | villous adenoma | |
| 278 | 37 | 5 | W | B |
| 130 | 17 | 7 | M | D |
| 265 | 0 (651)[a] | 0 (43) | M | A |
| 263 | 0 (809) | 1 (52) | M | A |

[a]Figures in parenthesis refer to MLF obtained on fixed cells; [b] W – well differentiated. M – moderately differentiated. P – poorly differentiated; [c]ND = not determined.

**Table II** Expression of HLA-DR as recognised by RF-B-HLA-DR monoclonal antibody by immunofluorescence on disaggregated cells from colorectal tumours

| *Immunofluorescence of RF-B-HLA-DR* | | | *Clinicopathology* | |
|---|---|---|---|---|
| *Tumour* | *MLF* | *% of cells stained* | *Differentiation*[a] | *Stage* |
| 125 | 820 | 69 | M | D |
| 299 | 667 | 94 | M | C |
| 279 | 510 | 37 | P | B |
| 295 | 454 | 83 | P | B |
| 302 | 353 | 63 | W | A |
| 290 | 253 | 52 | M | D |
| 298 | 230 | 70 | M | D |
| 294 | 222 | 51 | P | B |
| 316 | 204 | 29 | M | B |
| 275 | 180 | 28 | P | D |
| 296 | 177 | 26 | M | A |
| 301 | 176 | 32 | W | C |
| 318 | 163 | 35 | P | B |
| 323 | 145 | 15 | M | B |
| 317 | 109 | 18 | M | B |
| 310 | 88 | 15 | M | D |
| 283 | 85 | 22 | M | D |
| 315 | 60 | 11 | M | B |
| 314 | 55 | 9 | villous adenoma | |
| 312 | 55 | 6 | M | B |
| 281 | 45 | 6 | M | C |
| 303 | 45 | ND[b] | M | D |
| 278 | 35 | 7 | W | B |
| 277 | 33 | 6 | M | A |
| 282 | 26 | 8 | villous adenoma | |
| 287 | 0 | 0 | villous adenoma | |

[a]W – well differentiated. M – moderately differentiated. P – poorly differentiated; [b]ND = not determined.

Only three (12%) of the tumours stained with RF-B-HLA-DR in the range of MLF of 500–1,000, 12 (46%) tumours stained below a MLF of 500 and 11 (42%) failed to stain (MLF <100). Although all of the poorly differentiated tumours expressed HLA-DR there was no correlation between the intensity of staining and tumour differentiation. There was no correlation with expression of HLA-DR and clinicopathological stage.

Twenty-four percent of colorectal tumours stained heterogeneously with monoclonal antibody W6/32 (25–74% of cells/tumour stained). Thirty-three percent of these were poorly differentiated tumours whereas only 24% of the tumours, where greater than 75% of the cells/tumour stained, were poorly differentiated. None of the four well differentiated tumours stained heterogeneously although only 68% and 30% of the cells of two of the three villous adenomas stained with W6/32.

Fifty percent of the tumours stained with monoclonal antibody RF-B-HLA-DR. Eighty-five percent of these tumours stained heterogeneously and were either well,

**Table III** Expression of HLA-ABC and HLA-DR antigens as recognised by W6/32 and RF-B-HLA-DR monoclonal antibody by immunofluorescence staining of disaggregated cells from primary and secondary colorectal tumours from the same patient

| *Immunofluorescence of W6/32 on:* | | | | |
|---|---|---|---|---|
| | *Primary tumours* | | *Secondary tumours* | |
| *Tumours* | *MLF* | *% of cells stained* | *MLF* | *% of cells stained* |
| 238 | 1,574 | 92 | 1,060[a] | 53 |
| 299 | 1,558 | 98 | 2,913[a] | 99 |
| 240 | 938 | 74 | 653 | 63 |
| 275 | 720 | 62 | 736 | 70 |
| 310 | 506 | 76 | 1,420 | 89 |
| 303 | 346 | ND[b] | 66 | ND |
| 242 | 241 | 56 | 2,248[a] | 94 |
| 242 | 241 | 56 | 216 | 68 |
| 130 | 17 | 7 | 33 | 14 |
| *Immunofluorescence of RF-B-HLA-DR on:* | | | | |
| | *Primary tumours* | | *Secondary tumours* | |
| *Tumours* | *MLF* | *% of cells stained* | *MLF* | *% of cells stained* |
| 299 | 667 | 94 | 1,070[a] | 96 |
| 275 | 180 | 28 | 149 | 20 |
| 310 | 88 | 15 | 235 | 39 |
| 303 | 45 | ND | 13 | ND |

[a]Secondary tumour cells isolated from large hardened draining lymph nodes. All other tumour cells were isolated from liver metastases; [b]ND = not determined.

moderately or poorly differentiated and from all clinicopathological stages.

Secondary tumours were obtained from nine patients. Table III shows the intensity and percentage of cells staining with W6/32 and RF-B-HLA-DR for each secondary and its autologous primary tumour. There was no clear relationship between the primary and autologous secondary tumours with respect to either the intensity of cell surface staining or the percentage of cells recognised.

*Expression of HLA-ABC and HLA-DR on cryopreserved tumour sections*

Tumours staining with varying intensities by immunofluorescence (MLF 293-1574) on disaggregated tumour cells were also immunoperoxidase stained as cryopreserved tumour sections (Table IV). The variation in intensity of staining of W6/32 monoclonal antibody by immunofluorescence and FACS IV analysis was not observed in the immunoperoxidase stained sections. Six of the ten tumours stained strongly immunohistochemically whilst the remaining four stained moderately. Sections from two tumours stained with W6/32 immunohistochemically but failed to stain by immunofluorescence on fresh cells. However, when the cells from one of these tumours was fixed, strong intracellular immunofluorescence staining was observed. Staining with the RF-B-HLA-DR monoclonal antibody correlated for the two types of staining. Tumour sections staining moderately, stained moderately by immunofluorescence on fresh cells (MLFs 820-454) whereas tumours staining weakly by immunoperoxidase staining of tumour sections stained weakly by immunofluorescence (MLF 33-225). However the variation in intensities between individual tumours was much clearer by FACS IV analysis of freshly stained cells.

Immunohistochemical staining of normal large bowel showed uniform staining with W6/32 monoclonal antibody and no HLA-DR staining (data not shown).

**Table IV** Expression of HLA-ABC or HLA-DR as recognised by W6/32 and RF-B-HLA-DR on cryopreserved tumour sections or on disaggregated tumour cells

| Staining with W6/32 | | |
|---|---|---|
| *Tumour* | *Immunochemistry (cryopreserved sections)* | *Immunofluorescence (MLF) (fresh tumour cells)* |
| 238 | 2+[a] | 1,574 |
| 264 | 2+ | 1,546 |
| 125 | 2+ | 1,381 |
| 250 | + | 996 |
| 249 | + | 730 |
| 283 | 2+ | 569 |
| 266 | 2+ | 514 |
| 277 | + | 293 |
| 263 | + | 0 |
| 265 | 2+ | 0 (651) |
| *Staining with RF-B-HLA-DR* | | |
| *Tumour* | *Immunochemistry (cryopreserved sections)* | *Immunofluorescence (MLF) (fresh tumour cells)* |
| 125 | + | 820 |
| 299 | + | 667 |
| 279 | + | 510 |
| 295 | + | 454 |
| 298 | ± | 225 |
| 283 | ± | 85 |
| 277 | ± | 33 |

[a]2+ strong, +moderate, ±weak.

**Table V** Expression of MHC and tumour associated antigens in newly derived colorectal tumour cell lines

| | *Immunofluorescence with monoclonal antibodies (MLF):* | | | |
|---|---|---|---|---|
| *Culture* | *W6/32* | *RF-B-HLA-DR* | *C14* | *791T/36* |
| C146 | 0 (320) | 7 (155) | 3,242 | 377 |
| C168 | 11 (538) | 108 (1,036) | 2,195 | 184 |
| C170 | 5 (42) | 7 (155) | 2,298 | 185 |
| 223 | 0 (354) | 0 (156) | 1,274 | 532 |
| 224 | 0 (554) | 0 (920) | 549 | 478 |
| 225 | 0 (351) | 31 (750) | 479 | 138 |
| 277 | 378 (524) | 10 (10) | 278 | 798 |
| 280 | 408 (525) | 9 (56) | 765 | 300 |

Figures in parenthesis refer to MLF values obtained following paraformaldehyde fixation.

*Expression of MHC antigens on colorectal cells growing in early* in vitro *culture*

In contrast to the primary tumours, where 90% stained with monoclonal antibody W6/32, only two of the eight tumours which grew *in vitro* expressed HLA-ABC antigens at their cell surface. However, seven out of eight of these cultures expressed internal HLA-ABC antigens which were detected in fixed but not fresh cells (Table V). Only one of the cell lines expressed HLA-DR on its membrane whereas 50% of the primary tumours reacted with the RF-B-HLA-DR monoclonal antibody. However, 75% of these cultures expressed internal HLA-DR antigen which could be detected by RF-B-HLA-DR in fixed cells. (Table V). Furthermore two of the cell lines, C170 and C168 could be induced to express HLA-DR at their cell surface (Figure 1).

All of the early *in vitro* cultures expressed the tumour associated antigens defined by the monoclonal antibodies C14 and 791T/36 (Table V).

**Figure 1** Expression of HLA-ABC and HLA-DR as recognised by monoclonal antibodies W6/32 and RF-B-HLA-DR in C170 cells in the presence of human recombinant γIFN. (●) C170 cells stained with RF-B-HLA-DR, (○) C170 cells stained with W6/32, (■) C168 cells stained with RF-B-HLA-DR, (□) C168 cells stained with W6/32.

**Table VI** Expression of HLA-ABC and HLA-DR antigens as recognised by W6/32 and RF-B-HLA-DR monoclonal antibodies in an immunofluorescence assay on collagenase disaggregated cells from a series of adenocarcinomas

| *Immunofluorescence* | | | | | | | |
|---|---|---|---|---|---|---|---|
| *Aneuploid* | | | | *Diploid* | | | |
| *W6/32* | | *RF-B-HLA-DR* | | *W6/32* | | *RF-B-HLA-DR* | |
| *MLF* | *% positive* | *MLF* | *% positive* | *MLF* | *% positive* | *MLF* | *% positive* |
| 1,574 | 92 | ND[a] | ND | 730 | 75 | ND | ND |
| 938 | 74 | ND | ND | 996 | 92 | ND | ND |
| 241 | 56 | ND | ND | 706 | 62 | ND | ND |
| 700 | 84 | 45 | 5 | 1,546 | 92 | ND | ND |
| 569 | 83 | 85 | 20 | 0 | 0 | 0 | 0 |
| 1,590 | 97 | 222 | 51 | 720 | 62 | 180 | 28 |
| 564 | 95 | 454 | 83 | 293 | 50 | 33 | 6 |
| 1,558 | 98 | 667 | 94 | 812 | 92 | 126 | 8 |
| 2,327 | 99 | 176 | 32 | 1,585 | 96 | 177 | 8 |
| 3,110 | 98 | 353 | 63 | 153 | 27 | 353 | 63 |
| 506 | 76 | 88 | 15 | 191 | 75 | 88 | 15 |
| 479 | 75 | 60 | 10 | 713 | 61 | 60 | 10 |
| 688 | 87 | 204 | 29 | 545 | 68 | 55 | 6 |

[a]ND = not determined.

**Table VII** Expression of MHC and tumour associated antigens in colorectal cancer

| *% of tumours co-expressing MHC antigens and tumour associated antigens* | | | |
|---|---|---|---|
| *Tumours co-staining by immunofluorescence with the following monoclonal antibodies* | *Immunofluorescence with W6/32:* | | |
| | *100–75% of cells/ tumour stained* | *74–25% of cells/ tumour stained* | *<25% of cells/ tumour stained* |
| C14, 365, 791T/36 | 73 | 50 | 0 |
| C14, 365 | 16 | 12.5 | 0 |
| C14, 791T/36 | 0 | 12.5 | 0 |
| C14 | 11 | 25 | 33 |
| 365 | 0 | 0 | 67 |
| *Tumours co-staining by immunofluorescence with the following monoclonal antibodies* | *Immunofluorescence with RF:B:HLA-DR:* | | |
| | *100–75% of cells/ tumour stained* | *74–25% of cells/ tumour stained* | *<25% of cells/ tumour stained* |
| C14, 365, 791T/36 | 100 | 75 | 52 |
| C14, 365 | 0 | 12.5 | 16 |
| C14 | 0 | 12.5 | 16 |
| 365 | 0 | 0 | 16 |

### *Expression of MHC antigens in relation to DNA ploidy*

The aneuploid tumours bound W6/32 and RF-B-HLA-DR monoclonal antibodies with significantly stronger intensities ($t=2.4$; $P<0.05$; $t=2.5$; $P<0.05$) than diploid tumours (Table VI). Only one of the aneuploid tumours stained heterogeneously whereas over half of the diploid tumours showed this patchy expression. Eighty percent of the aneuploid tumours expressed HLA-DR whereas only 27% of diploid tumours expressed this antigen.

### *Expression of HLA-ABC and HLA-DR antigens in relation to tumour associated antigens*

Seventy-three percent of the tumours, in which >75% of the cells expressed HLA-ABC, also expressed the epitopes defined by monoclonal antibodies C14, 365 and 791T/36 (Table VII). Only 50% of the tumours staining heterogeneously, and none of the tumours failing to stain with monoclonal antibody W6/32, co-expressed the three tumour associated antigens. One hundred percent of the tumours which failed to express HLA-ABC only co-expressed one tumour associated antigen. HLA-ABC was never expressed on its own (Table VII).

There was no correlation between the intensity of staining with W6/32 monoclonal antibody and the monoclonal antibodies RF-B-HLA-DR, C14, 365 and 791T/36 (Table VIII).

All of the tumours in which >75% of the cells stained with RF-B-HLA-DR co-expressed the epitopes defined by monoclonal antibodies C14, 365 and 791T/36. Seventy-five percent of tumours staining heterogeneously with monoclonal antibody RF-B-HLA-DR and 52% of the tumours which failed to stain, also stained with monoclonal antibodies C14, 365 and 791T/36. Thirty-two percent of the tumours which failed to express HLA-DR antigen only co-expressed one of the tumour associated antigens. HLA-DR antigen was never expressed on its own.

**Table VIII** Expression of HLA-ABC and HLA-DR antigens in association with carcinoembryonic antigen, 791T p72 and Y hapten blood group as recognised by the monoclonal antibodies W6/32, RF-B-HLA-DR, C14, 365, 791T/36

| | *Immunofluorescence staining with monoclonal antibodies (MLF)* | | | | |
|---|---|---|---|---|---|
| *Tumour* | *W6/32* | *RF-B-HLA-DR* | *C14* | *365* | *791T/36* |
| 302 | 3,110 | 353 | 1,778 | 225 | 25 |
| 301 | 2,327 | 176 | 793 | 1,276 | 300 |
| 294 | 1,590 | 222 | 525 | 1,638 | 586 |
| 296 | 1,586 | 177 | 515 | 1,025 | 466 |
| 238 | 1,574 | ND[a] | 406 | 832 | 655 |
| 299 | 1,558 | 667 | 1,744 | 1,471 | 1,278 |
| 264 | 1,546 | ND | 1,453 | 1,042 | 133 |
| 125 | 1,381 | 820 | 1,430 | 506 | 366 |
| 262 | 1,225 | ND | 671 | 936 | 40 |
| 142 | 1,078 | ND | 1,179 | 866 | 463 |
| 248 | 958 | ND | 1,158 | 50 | 162 |
| 290 | 849 | 253 | 280 | 350 | 240 |
| 282 | 812 | 26 | 159 | 61 | 17 |
| 279 | 809 | 510 | 1,122 | 65 | 83 |
| 317 | 789 | 109 | 563 | 281 | 104 |
| 298 | 777 | 230 | 927 | 1,414 | 324 |
| 275 | 720 | 180 | ND | 1,912 | 237 |
| 312 | 713 | 55 | 404 | 272 | 111 |
| 281 | 700 | 45 | 166 | 400 | 157 |
| 316 | 688 | 204 | 466 | 317 | 140 |
| 318 | 670 | 163 | 1,516 | 1,529 | 609 |
| 323 | 648 | 145 | 1,909 | 764 | 323 |
| 283 | 569 | 85 | 1,397 | 1,740 | 123 |
| 295 | 564 | 454 | 126 | 247 | 152 |
| 314 | 545 | 55 | 107 | 200 | 43 |
| 266 | 514 | ND | 571 | 677 | 5 |
| 310 | 506 | 88 | 470 | 847 | 319 |
| 315 | 479 | 60 | 312 | 403 | 85 |
| 241 | 453 | ND | 217 | 618 | 566 |
| 303 | 346 | 45 | 145 | 158 | 194 |
| 277 | 293 | 33 | 73 | 148 | 21 |
| 242 | 241 | ND | 548 | 2,121 | 200 |
| 309 | 191 | 55 | 177 | 77 | 71 |
| 236 | 188 | ND | 1,661 | 2,020 | 1,423 |
| 287 | 141 | 0 | 240 | 51 | 28 |
| 278 | 37 | 35 | 20 | 478 | 52 |
| 265 | 0 | ND | 214 | 27 | 16 |
| 263 | 0 | ND | 114 | 0 | 0 |

[a]ND: not determined

### Discussion

The majority of nucleated cells express HLA-ABC antigens (Bodmer, 1981). Thirty-four percent of the colorectal tumours when analysed by a FACS IV cells sorter were partially or completely negative for cell surface HLA-ABC antigen expression. This agreed with the results of Csiba *et al.* (1984) who observed partial absence of class I antigens in 40% of their colorectal cancers. However, Momburg *et al.* (1986) only observed loss of HLA-ABC antigens in 13% of colorectal cancers analysed and Daar and Fabre (1983) observed loss of class I in only 1/15 of the colorectal cancers they studied. Tumours stained by immunohistochemistry are fixed prior to staining and therefore it is impossible to distinguish internal and external antigen expression. Interestingly two of the tumours which failed to express HLA/ABC could be stained with W6/32 monoclonal antibody following fixation. Similarly 6 of the 8 cultured cell lines only expressed internal HLA-ABC antigens. Negative results reflect abnormalities in the synthesis, assembly, insertion into the plasma membrane and for shedding of HLA-ABC antigens. Expression of only internal antigen in some primary tumours and cultured cell lines maybe suggests an abnormality in insertion into the plasma membrane. Biological behaviour, as measured by tumour growth and propensity to metastasise varies considerably between tumours of a given type. As the external membrane of tumours and all other cells dictates the nature of their interactions with their environment, membrane changes may be associated with tumour behaviour. This study was concerned with quantitative evaluation of cell surface MHC (and tumour associated antigen expression) as a potential marker of tumour progression.

There was an enormous variation in the intensity of staining with W6/32 monoclonal antibody which could not be detected by immunohistochemistry. The level of class I antigen expression may affect sensitivity to lysis by natural killer cells (Ljunggren & Karre, 1985). Studies with rat tumour cells indicated that the appearance of increased class I antigen induced by rat γIFN closely parallels changes in sensitivity to natural killer cells (Yeoman *et al.*, 1986).

Although there was no correlation between the intensity of staining with W6/32 monoclonal antibodies and either histological grade or clinicopathological stage A, it will be interesting to see if there is any subsequent correlation with patient survival. In the mouse T10 sarcoma model manipulations which resulted in increased class I antigen expression were associated with increased metastatic potential (Katzav *et al.*, 1983). This was related to high levels of H-2D expression whereas gene transfection studies in the same lines showed that increased H-2K gene expression resulted in variants with decreased metastatic activity. Furthermore, this effect was related to an immune response, as the same variants metastasized in immunodeprived recipients (Wallich *et al.*, 1985). These findings are consistent with the hypothesis that tumour associated antigens are recognised in the context of H-2K and not H-2D class I antigens. Further studies using monoclonal antibodies specific to each of the human class I loci will determine if any one MHC class locus is a better indicator of tumour aggression.

Epithelial cells do not usually express HLA-DR antigens however 50% of the colorectal tumours expressed this antigen. Although the intensity of staining varied enormously (range of MLFs of 0–810), the majority of tumours stained weakly (MLF <300). All of the poorly differentiated tumours expressed HLA-DR confirming the suggestion of Rognum *et al.* (1983) that HLA-DR expression is more consistent in poorly differentiated tumours. In agreement with previous studies (Daar & Fabre, 1983; Csiba *et al.*, 1984) there was no correlation between expression of HLA-DR antigen and clinicopathological stage. Expression of HLA-DR antigens on primary tumours can augment the immunogenicity of tumour associated antigens as they are important in antigen presentation to helper T-lymphocytes (Fossati *et al.*, 1984). However, metastatic melanoma cells expressing high levels of HLA-DR antigens can inhibit the immune response of autologous peripheral blood lymphocytes. Furthermore it appears that a T-lymphocyte-derived lymphokine such as γIFN can influence both the phenotype and the suppressive activity of autologous metastatic melanoma cells (Taramelli *et al.*, 1984).

Although Thompson *et al.* (1982) reported that metastatic colorectal tumours were consistently HLA-DR antigen negative, 2/4 of our secondary tumours expressed this antigen. However, it was found that the majority of early derived *in vitro* dividing cells were consistently negative for both HLA-ABC and HLA-DR antigens. However, re-expression of MHC antigens could be induced in several cell lines by the immune regulator γIFN. This could imply that antigen expression *in vivo* is induced by local γIFN, and the lack of expression *in vitro* is due to lack of γIFN. Alternatively if there is a correlation between *in vitro* and *in vivo* growth perhaps tumours are maintained and seed by cell surface MHC antigen negative cells which may escape immune recognition. Re-expression on maturation may be controlled by immune regulators such as γIFN.

In agreement with Rognum *et al.* (1982) the aneuploid tumours stained more homogeneously with RF-B-HLA-DR

and with a higher intensity than the diploid tumours. This study also showed a similar correlation with expression of HLA-ABC antigens. Abnormal expression of the tumour associated antigens CEA, Y haptenic blood group and 791T p72 also correlated with expression of HLA-ABC and HLA-DR antigens on colorectal tumours. Previous studies show that tumour associated antigens are also expressed more strongly on aneuploid than diploid tumours (Durrant *et al.*, 1986*a*). Perhaps gene amplification in aneuploid tumours results in increased antigen expression. Our group has previously shown that patients with aneuploid tumours have a significantly worse survival than patients with diploid tumours (Armitage *et al.*, 1985). Perhaps elevated HLA-class I expression is associated with increased metastatic potential as seen in animal models (Katzav *et al.*, 1983).

Prospective studies currently in progress should determine if the quantity of MHC antigens on human colorectal cancer correlates with tumour recurrence, and metastatic spread, allowing an early prediction of which stage B and C tumours are most aggressive.

These studies were supported by the Cancer Research Campaign, U.K. The skilful technical assistance of Mr O. Roberts and Miss J. Wright is gratefully acknowledged.

**References**

ARMITAGE, N.C., ROBINS, R.A., EVANS, D.F., TURNER, D., BALDWIN, R.W. & HARDCASTLE, J.D. (1985). The influence of tumour cell DNA content on survival in colorectal cancer. *Br. J. Surg.*, **72**, 828.

BARNSTABLE, C., BODMER, W., BROWN, G. & 4 others (1978). Production of monoclonal antibodies to group A erythrocytes HLA and other human cell surface antigens as new tools for genetic analysis. *Cell*, **4**, 9.

BENACERRAF, B. (1981). Role of MHC gene products in immune regulation. *Science*, **212**, 1229.

BODGER, M.P., IZAGUIRRE, C.A., BLACKLOCK, H.A. & HOFFBRAND, A.V. (1983). Surface antigenic determinants on human pluripotent and unipotent hematopoeitic progenitor cells. *Blood*, **61**, 1006.

BODMER, W.F. (1981). HLA structure and function: A contem porary view. *Tissue Antigens*, **17**, 19.

BROWN, A., FEIZI, T., GOOI, H.C., EMBLETON, M.J., PICARD, J.K. & BALDWIN, R.W. (1983). A monoclonal antibody against human colonic adenoma recognises a difucosylated Type-2 blood group chain. *Bioscience Rep.*, **3**, 163.

CSIBA, A., WHITWELL, H.L. & MOORE, M. (1984). Distribution of histocompatibility and leucocyte differentiation antigens in normal human colon and in benign and malignant colonic neoplasms. *Br. J. Cancer*, **50**, 699.

DALCHAU, R., KIRLEY, J. & FABRE, S.W. (1980). Monoclonal antibody to a human leucocyte specific membrane glycoprotein probably homolgous to the leucocyte common antigen of the rat. *Eur. J. Immunol.*, **10**, 737.

DAAR, A.S. & FABRE, J.W. (1983). The membrane antigens of human colorectal cancer cells: Demonstration with monoclonal antibodies of heterogeneity within and between tumours and of anomalous expression of HLA-DR. *Eur. J. Cancer Clin. Oncol.*, **19**, 209.

DUKES, C.E. (1932). The classification of cancer of the rectum. *J. Path. Bact.*, **35**, 323.

DURRANT, L.G., ROBINS, R.A., ARMITAGE, N.C., BROWN, A., BALDWIN, R.W. & HARDCASTLE, J.D. (1986*a*). Association of antigen expression and DNA ploidy in colorectal cancer. *Cancer Res.*, **46**, 3543.

DURRANT, L.G., ROBINS, R.A., PIMM, M.V. & 4 others (1986*b*). Antigenicity of newly established colorectal carcinoma cell lines. *Br. J. Cancer*, **53**, 37.

EMBLETON, M.J., GUNN, B., BYERS, V.S. & BALDWIN, R.W. (1981). Antitumour reaction of a monoclonal antibody against a human osteogenic sarcoma cell line. *Br. J. Cancer*, **43**, 582.

FOSSATI, G., TARAMELLI, D., BALSARI, A., BOGDANOVICH, S., ANDREOLA, A. & PARMIANI, G. (1984). Primary but not metastatic human melanomas expressing DR antigens stimulate autologous lymphocytes. *Int. J. Cancer*, **33**, 591.

HOLMES, C.H., AUSTIN, E.B., FISK, A., GUNN, B. & BALDWIN, R.W. (1984). Monoclonal antibodies reacting with normal rat liver cells as probes in hepatocarcinogenesis. *Cancer Res.*, **44**, 1611.

HUI, K., GROSVELD, F. & FESTENSTEIN, H. (1984). Rejection of transplantable AKR leukaemia cells following MHC DNA mediated cell transformation. *Nature*, **311**, 750.

KARRE, K., LJUNGGREN, H.G., PIONTEK, G. & KEISSLING, R. (1986). Selective rejection of H-2 deficient lymphomas variants suggests alternative immune defence strategy. *Nature*, **319**, 675.

KATZAV, S., DE BAETSELIER, P., TARTAKOVSKY, B., FELDMAN, M. & SEGAL, S. (1983). Alterations in major histocompatibility complex phenotypes of mouse cloned T10 sarcoma cells: Association with shifts from non metastatic to metastatic cells. *J. Natl Cancer Inst.*, **71**, 317.

LJUNGGREN, H.G. & KARRE, K. (1985). Host resistance directed selectively against H-2 deficient lymphoma variants. *J. Exp., Med.*, **162**, 1745.

LONAI, P., STEINMAN, L., FREDMAN, V., DRIZLIKH, G. & PURI, J. (1981). Specificity of antigen binding by T-cells: Competition between soluble and Ia-associated antigen. *Eur. J. Immunol.*, **11**, 382.

MCKENZIE, J.K.L. & FABRE, J.W. (1981). Human Thy 1 unusual localisation and possible functional significance in lymphoid tissues. *J. Immunol.*, **126**, 843.

MOMBURG, F., DEGENER, T., BACCHUS, E., MOLDENHAUER, G., HAMMERLING, G.J. & MOLLER, P. (1986). Loss of HLA-A, B, C and de novo expression of HLA-D in colorectal cancer. *Int. J. Cancer*, **37**, 179.

PRICE, M.R., CAMPBELL, D.G., ROBINS, R.A. & BALDWIN, R.W. (1983). Characteristics of a cell surface antigen defined by an anti-human osteogenic sarcoma monoclonal antibody. *Eur. J. Cancer Clin. Oncol.*, **19**, 81.

ROBINS, R.A. (1986). T-cell responses at the host:tumour interface. *Biochim. Biophys. Acta*, **865**, 289.

ROE, R., ROBINS, R.A., LAXTON, R.R. & BALDWIN, R.W. (1985). Kinetics of divalent monoclonal antibody binding to tumour cell surface antigens using flow cytometry: Standardization and mathematical analysis. *Molecular Immunology*, **22**, 11.

ROGNUM, T.O., BRANDTZAEG, P. & THORUD, E. (1983). Is heterogeneous expression of HLA-DR antigens and CEA along with DNA profile variations evidence of phenotypic instability and clonal proliferation in human bowel carcinomas? *Br. J. Cancer*, **48**, 543.

TARAMELLI, D., FOSSATI, G., BALSARI, A., MAROLDA, R. & PARMIANI, G. (1984). Inhibition of lymphocyte stimulation by autologous human metastatic melanoma cells correlates with the expression of HLA-DR antigens on the tumor cells. *Int. J. Cancer*, **34**, 797.

THOMSON, S.S., HERLYN, M.F., ELDER, D.E., CLERC, W.H., STEPLEWSKY, Z. & KOPROWSKI, H. (1982). Expression of DR antigens in freshly frozen human tumors. *Hybridoma*, **1**, 161.

WALLICH, R., BULBUC, N., HAMMERLING, G.J., KATZAV, S., SEGAL, S. & FELDMAN, M. (1985). Abrogation of metastatic properties of tumour cells by de novo expression of H-2K antigens following H-2 gene transfection. *Nature*, **315**, 301.

WHITWELL, H.L., HUGHES, H.P.A., MOORE, M. & AHMED, A. (1984). Expression of major histocompatibility antigens and leucocyte infiltration in benign and malignant human breast disease. *Br. J. Cancer*, **49**, 161.

YEOMAN, H. & ROBINS, R.A. (1986). The effect of interferon gamma treatment of rat tumour cells on their susceptibility to natural killer cell, macrophages and cytotoxic T-cell killing. *Imunology* (in press).

ZINKERNAGEL, R.M. & DOHERTY, P.C. (1979). MHC cytotoxic T-cells: studies on the biological role of polymorphic major transplantation antigens determining T-cell restriction-specificity, function and responsiveness. *Adv. Immunol.*, **27**, 51.